# Routine Blood Indicators Combined With Sleep Questionnaires for the Identification of Cognitive Impairment in Parkinson's Disease: A Cross‐Sectional Study With a Leakage‐Free Nested Modeling Framework

**DOI:** 10.1002/brb3.71655

**Published:** 2026-09-07

**Authors:** Ziyi Wang, TszLeong Lam, Shuo Zhang, Chuanjin Song, Xiaoxue Pei, Junhong Zhong, Bin Li, Guilin Liu, Yingxue Cui, Shaosong Wang

**Affiliations:** ^1^ Beijing University of Chinese Medicine Beijing China; ^2^ Capital Medical University Beijing China; ^3^ Department of Acupuncture, Beijing Hospital of Traditional Chinese Medicine Capital Medical University Beijing China

**Keywords:** blood indicators, cognitive impairment, cross‐sectional study, nested logistic regression, Parkinson's disease

## Abstract

**Background:**

Cognitive impairment (CI) is a prevalent non‐motor complication of Parkinson's disease (PD), yet accessible and reliable screening aids remain limited. This cross‐sectional study aimed to quantify the strength of association and the cross‐sectional classification value of routine blood indicators combined with standardized sleep questionnaires for prevalent CI in PD, using a strictly leakage‐free model‐development pipeline.

**Methods:**

A total of 347 PD patients were enrolled and classified into PD‐CI (*n* = 143) and PD‐NCI (*n* = 204) groups based on the Montreal Cognitive Assessment (MoCA < 26). The cohort was first divided into training (70%, *n* = 242) and test (30%, *n* = 105) sets by stratified sampling; all subsequent feature selection and tuning were confined to the training data. Within the training set, 20 candidate predictors were screened using L1‐penalized logistic regression with the lambda.1se criterion, yielding 10 variables. Four nested logistic regression models (baseline clinical, baseline plus blood, baseline plus sleep, and full model) were constructed alongside random forest (RF) and gradient boosting machine (GBM) classifiers. Discrimination was evaluated by the area under the receiver operating characteristic curve (AUC) with DeLong confidence intervals and between‐model DeLong tests, calibration (slope, intercept, and Brier score with bootstrap intervals) and decision curve analysis; the untouched test set was scored once, and generalization was estimated by repeated 5 × 2 cross‐validation. Robustness of the outcome definition was examined with education‐adjusted and alternative MoCA cut‐offs.

**Results:**

The training‐set LASSO retained 10 predictors: education, disease duration, Hoehn–Yahr stage, platelet‐to‐lymphocyte ratio (PLR), monocyte‐to‐lymphocyte ratio (MLR), homocysteine, C‐reactive protein (CRP), Pittsburgh Sleep Quality Index (PSQI), Epworth Sleepiness Scale (ESS), and REM Sleep Behavior Disorder Screening Questionnaire (RBDSQ). In the untouched test set, the full logistic regression model achieved an AUC of 0.943 (95% CI: 0.896–0.990) in the test set, higher than baseline (0.844), and comparable to the baseline‐plus‐blood (0.937) and baseline‐plus‐sleep (0.889) models; the incremental gain over baseline was statistically significant (DeLong *p* = 0.009). Repeated cross‐validation gave a more conservative AUC of 0.921 (SD = 0.029). RF (0.956) and gradient boosting (0.944) did not outperform logistic regression. Calibration was acceptable (slope 1.18, intercept 0.03; test Brier 0.086, 95% CI 0.057–0.120) and findings were stable across alternative MoCA cut‐offs (AUC 0.909–0.931).

**Conclusion:**

Combining routine blood indicators and sleep questionnaires is cross‐sectionally associated with prevalent CI in PD and adds information beyond clinical variables alone. Because the design is cross‐sectional, single‐center, and only internally validated, the model should be regarded as a candidate screening aid that requires external, preferably multicenter and prospective, validation before any clinical use.

## Graphical abstract

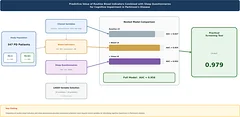

## 1 Introduction

Parkinson's disease (PD) is a progressive neurodegenerative disorder characterized by motor symptoms including bradykinesia, rigidity, and resting tremor, as well as a broad spectrum of non‐motor features (Poewe et al. 2017). Among these non‐motor manifestations, cognitive impairment (CI) stands out as one of the most debilitating complications, with reported prevalence rates ranging from 20% to 57% depending on the diagnostic criteria applied and the population studied (Aarsland et al. 2017; Litvan et al. 2012). Cognitive decline in PD encompasses a continuum from mild cognitive impairment (PD‐MCI) to Parkinson's disease dementia (PDD), and its presence is strongly associated with reduced quality of life, increased caregiver burden, higher healthcare costs, and accelerated disease progression (Aarsland et al. 2021; Lawson et al. 2014). Early identification of PD patients with or at risk for CI is therefore of paramount clinical importance, as it enables timely intervention and targeted management strategies. Neuropsychological assessments such as the Montreal Cognitive Assessment (MoCA) and Mini‐Mental State Examination (MMSE) are commonly employed for screening, but they are time‐consuming, influenced by educational background, and subject to practice effects (Nasreddine et al. 2005). As a result, there is a growing demand for objective, easily accessible biomarkers that can complement or enhance traditional cognitive screening approaches.

Peripheral blood indicators have attracted considerable interest as candidate biomarkers for neurodegeneration. Systemic inflammatory markers, including the neutrophil‐to‐lymphocyte ratio (NLR), platelet‐to‐lymphocyte ratio (PLR), monocyte‐to‐lymphocyte ratio (MLR), and C‐reactive protein (CRP), reflect chronic low‐grade inflammation that has been implicated in the pathogenesis of both PD and cognitive decline (Qin et al. 2016; Gao and Hong 2008). Elevated levels of homocysteine (Hcy), a sulfur‐containing amino acid, have been consistently linked to neuronal toxicity, vascular endothelial dysfunction, and an increased risk of dementia in PD (Sleeman et al. 2019; Sapkota et al. 2021). Conversely, serum albumin (ALB) and uric acid (UA), which possess antioxidant properties, have been reported to exhibit protective associations with cognitive function in neurodegenerative diseases (Ascherio et al. 2009; Sun et al. 2022). In addition, metabolic parameters such as fasting glucose and glycated hemoglobin (HbA1c) are relevant given the well‐documented association between insulin resistance and cognitive decline (Bosco et al. 2012). While individual blood markers have shown promising associations with PD‐CI in cross‐sectional and longitudinal studies, the discriminative performance of single biomarkers is generally modest, prompting researchers to explore multivariable models that combine multiple indicators.

Sleep disturbances represent another category of non‐motor symptoms that are both highly prevalent in PD and closely interrelated with cognitive function. The Pittsburgh Sleep Quality Index (PSQI), Epworth Sleepiness Scale (ESS), REM Sleep Behavior Disorder Screening Questionnaire (RBDSQ), and Insomnia Severity Index (ISI) capture distinct dimensions of sleep pathology, including global sleep quality, daytime somnolence, REM sleep behavior disorder (RBD), and insomnia severity, respectively (Buysse et al. 1989; Johns 1991). RBD is of particular interest because it is considered a prodromal marker of synucleinopathy and has been independently associated with the development of dementia in PD (Postuma et al. 2019; Fereshtehnejad et al. 2015). Poor sleep quality and excessive daytime sleepiness may also accompany impaired cognitive performance through disruption of memory consolidation, reduced attention, and altered cerebral clearance of neurotoxic proteins via the glymphatic system (Xie et al. 2013; Diekelmann and Born 2010). Despite these well‐established pathophysiological links, few studies have systematically examined the joint association of sleep questionnaires and blood biomarkers with PD‐CI, and even fewer have employed rigorous variable selection and leakage‐free model validation methodologies.

The present study addresses these gaps by examining, in a cross‐sectional design, how routine blood indicators and standardized sleep questionnaires are associated with prevalent CI in PD and how much classification information they add beyond clinical variables. Because predictors and outcome were measured contemporaneously, we frame the work as an assessment of cross‐sectional associations and concurrent classification rather than temporal prediction, and we use the term “candidate model” throughout. Unlike previous investigations that simply combined all available predictors without formal feature selection, we applied L1‐penalized logistic regression (LASSO) with the one‐standard‐error rule (lambda.1se) to identify a parsimonious subset of informative variables. Critically, the cohort was partitioned into training and test sets before any feature selection or tuning, and LASSO and all hyperparameter tuning were performed exclusively within the training data (and re‐derived inside every cross‐validation fold), so that the test set could not influence model development (Kaufman et al. 2012; Varma and Simon 2006). We then employed a nested model comparison framework, constructing four sequential logistic regression models (baseline clinical variables, baseline plus blood indicators, baseline plus sleep scores, and the full combined model) to quantify the incremental classification value contributed by each predictor domain. In addition to logistic regression, we trained random forest (RF) and gradient boosting machine (GBM) classifiers to evaluate whether more flexible algorithms could improve discrimination. Model performance was assessed using receiver operating characteristic (ROC) analysis with DeLong tests, calibration (slope, intercept, and Brier score with bootstrap intervals), and decision curve analysis (DCA), with internal validation through a single held‐out test set and repeated cross‐validation; the robustness of the MoCA‐based outcome definition to education was examined in pre‐specified sensitivity analyses. Variance inflation factors (VIF) were calculated to address multicollinearity. This comprehensive analytical strategy aims to provide robust evidence regarding the clinical relevance of combining blood and sleep assessments for PD‐CI identification.

## 2 Methods

### 2.1 Study Design and Participants

**FIGURE 1:**
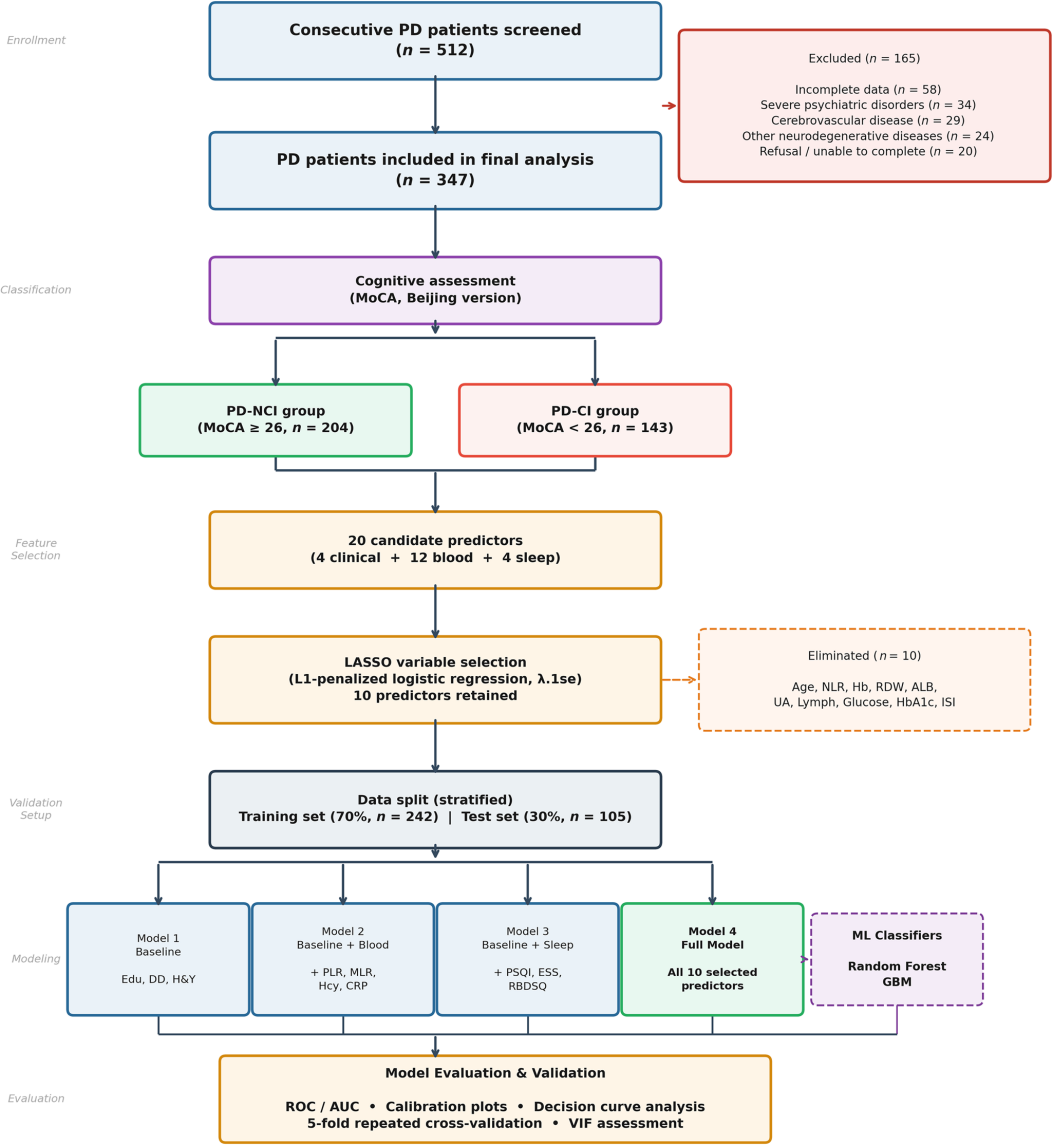
Study flow diagram (leakage‐free model‐development pipeline). A total of 512 patients with idiopathic Parkinson's disease (PD) were initially screened. After applying exclusion criteria (incomplete data, severe psychiatric disorders, cerebrovascular diseases, other neurodegenerative conditions, or refusal to participate), 347 patients were enrolled. Based on the Montreal Cognitive Assessment (MoCA) score, patients were classified into PD‐CI (MoCA < 26, *n* = 143) and PD‐NCI (MoCA ≥ 26, *n* = 204) groups. The cohort was then split into training (70%, *n* = 242) and test (30%, *n* = 105) sets before any feature selection or tuning (Step 1). L1‐penalized logistic regression (LASSO, *λ*.1se) and all model tuning were performed within the training data only (Step 2), reducing 20 candidates to 10 predictors; the test set was scored once at evaluation (Step 4). This ordering eliminates the information leakage present when selection precedes splitting.

This cross‐sectional study enrolled 512 consecutive patients diagnosed with idiopathic PD according to the Movement Disorder Society (MDS) clinical diagnostic criteria (Postuma et al. 2015) at the Department of Neurology, Beijing Hospital of Traditional Chinese Medicine, Capital Medical University, between January 2021 and December 2023. Because predictors (blood indicators, sleep questionnaires, and clinical variables) and the cognitive outcome were ascertained at the same visit, the study has no temporal separation between exposures and outcome; the analyses therefore estimate cross‐sectional associations and concurrent classification accuracy rather than prognostic prediction. Exclusion criteria were: (1) incomplete clinical or laboratory data (*n* = 58); (2) comorbid severe psychiatric disorders including major depressive disorder or psychosis requiring medication adjustment (*n* = 34); (3) history of stroke or other cerebrovascular diseases confirmed by neuroimaging (*n* = 29); (4) diagnosis of other neurodegenerative diseases such as Alzheimer's disease, multiple system atrophy, or progressive supranuclear palsy (*n* = 24); and (5) refusal to participate or inability to complete all assessments (*n* = 20). After exclusions, 347 patients were included in the final analysis (Figure 1). The study protocol was approved by the institutional ethics committee, the Ethics Committee of Beijing Hospital of Traditional Chinese Medicine, Capital Medical University (approval number #2018‐01), and all participants provided written informed consent.

The sample size was evaluated against the events per variable (EPV) criterion recommended for prediction model development (Riley et al. 2020; van Smeden et al. 2016). With 143 events (PD‐CI cases) and 10 final predictors selected by LASSO, the EPV was approximately 14.3, exceeding the minimum recommended threshold of 10 and indicating adequate statistical power for stable coefficient estimation (Peduzzi et al. 1996). Because feature selection and model fitting were restricted to the training set (*n* = 242, 100 events), the training‐set EPV was approximately 10.0; this is acknowledged as a limitation that further motivates external validation.

### 2.2 Cognitive Assessment and Group Classification

All participants underwent standardized cognitive assessment using the MoCA (Beijing version) and the MMSE administered by trained neuropsychologists blinded to laboratory results. CI was defined as a MoCA score below 26 points, consistent with established cut‐off values validated in Chinese PD populations (Yu et al. 2012). Participants with MoCA scores of 26 or above were classified into the PD‐NCI (no CI) group. We acknowledge that MoCA performance is strongly influenced by education and that a single fixed cut‐off may over‐classify impairment in less‐educated participants; because education is also a predictor, this could in principle inflate apparent performance through a measurement artifact (Litvan et al. 2012; Nasreddine et al. 2005; Yu et al. 2012; Lu et al. 2011). To address this, education was modeled explicitly, and the outcome definition was interrogated in four pre‐specified sensitivity analyses (Section 2.8): (1) an education‐adjusted MoCA threshold (adding one point for participants with ≤ 6 years of formal schooling, per Chinese validation studies (Lu et al. 2011)); (2) a stricter cut‐off of < 25; (3) a cut‐off of < 24; and (4) an operationalization approximating MDS Level II PD‐MCI criteria (Litvan et al. 2012). It should be noted that MoCA and MMSE scores served exclusively as the outcome classification criterion and were not included as predictor variables in any statistical model, thereby avoiding circular reasoning (Moons et al. 2015).

### 2.3 Blood Indicator Assessment

Fasting venous blood samples were collected between 7:00 and 9:00 a.m. on the morning following admission. Complete blood count parameters were analyzed using an automated hematology analyzer (Sysmex XN‐9000, Kobe, Japan), from which the following derived ratios were calculated: NLR, PLR, and MLR. Additional laboratory measurements included hemoglobin (Hb), red cell distribution width (RDW), ALB, UA, Hcy, high‐sensitivity CRP (hs‐CRP), lymphocyte count, fasting glucose, and HbA1c. All laboratory assays were performed according to standardized protocols with appropriate internal and external quality controls.

### 2.4 Sleep Questionnaire Assessment

Sleep disturbances were assessed using four validated self‐report questionnaires administered on the same day as cognitive testing. The PSQI evaluates global sleep quality over the preceding month across seven domains, with total scores ranging from 0 to 21 (higher scores indicating worse sleep quality) (Mollayeva et al. 2016). The ESS measures the propensity for daytime sleepiness using eight situational items scored from 0 to 24 (Johns 1992). The RBDSQ is a 13‐item screening instrument for RBD, with scores ranging from 0 to 13 (Stiasny‐Kolster et al. 2007). The ISI assesses the severity of insomnia symptoms over the past two weeks using seven items scored from 0 to 28 (Bastien et al. 2001). All questionnaires have been validated in Chinese populations and demonstrated acceptable psychometric properties in PD patients.

### 2.5 Statistical Analysis

Continuous variables were expressed as mean and standard deviation (SD) or median and interquartile range (IQR) depending on the distribution assessed by the Shapiro–Wilk test. Between‐group comparisons were performed using the independent samples *t*‐test or Mann–Whitney *U* test for continuous variables, and the chi‐square test for categorical variables. Spearman rank correlation coefficients were computed among blood indicators and sleep questionnaire scores. All correlation coefficients are reported as the exact values obtained, rounded to two decimal places; no thresholding, masking, or rounding to zero was applied (the previous heatmap had suppressed small coefficients, which is corrected in the revised Figure 4). No missing values were present in the 347 included patients, as incomplete data constituted a pre‐specified exclusion criterion. Differences in AUC between nested models were tested with the DeLong test for two correlated ROC curves (DeLong et al. 1988), in addition to reporting DeLong‐based 95% confidence intervals. All analyses were conducted in Python 3.10 (scikit‐learn 1.3, statsmodels 0.14, NumPy, SciPy) with a fixed random seed (20260612) for all stochastic steps; statistical significance was set at a two‐sided alpha of 0.05.

### 2.6 LASSO Variable Selection

To identify the most informative predictors from the 20 candidate variables (4 clinical, 12 blood, and 4 sleep), we employed LASSO with 10‐fold stratified cross‐validation (Tibshirani 1996). To prevent information leakage, the data were first split into training and test sets (Section 2.8); LASSO was then fitted exclusively on the training set, and the regularization path and the lambda.1se choice were determined by 10‐fold cross‐validation restricted to the training data. No information from the test set entered the selection step (Kaufman et al. 2012; Varma and Simon 2006). The regularization parameter lambda was selected using the one‐standard‐error rule (lambda.1se), which chooses the most parsimonious model within one standard error of the minimum cross‐validated log‐loss, rather than the lambda.min criterion (Friedman et al. 2010). This approach yields a sparser model with enhanced generalizability. All predictors were standardized to zero mean and unit variance using training‐set means and SDs, which were then applied unchanged to the test set. Variables with absolute LASSO coefficients exceeding 0.01 at the optimal lambda were retained for subsequent model construction. The procedure yielded 10 variables from the original 20. To assess selection stability under a fully leakage‐free protocol, the entire LASSO selection was additionally re‐derived inside each fold of a repeated 5 × 2 cross‐validation, and the selection frequency of every candidate was recorded (Table S2).

### 2.7 Model Construction and Nested Comparison

To evaluate the incremental classification value of blood indicators and sleep questionnaires beyond baseline clinical variables, four nested logistic regression models were constructed: Model 1 (Baseline) included only clinical variables (education, disease duration, and Hoehn–Yahr stage); Model 2 (Baseline + Blood) added LASSO‐selected blood indicators (PLR, MLR, Hcy, and CRP); Model 3 (Baseline + Sleep) added LASSO‐selected sleep variables (PSQI, ESS, and RBDSQ); and Model 4 (Full) included all 10 LASSO‐selected predictors. This nested design enables fair comparison of predictor domains with equivalent baseline adjustment (Steyerberg and Vergouwe 2014). In addition, RF and GBM classifiers were trained on the full selected variable set. All models, including the RF and GBM hyperparameters, were fitted and tuned using the training data only; the test set was never used for any tuning decision. After cross‐validated tuning on the training set, RF was configured with 400 trees, maximum depth 4, minimum leaf size 20, and √*p* features per split, and GBM with 120 trees, maximum depth 2, learning rate 0.05, and subsample fraction 0.7. The class distribution was preserved by stratification (training: 100 PD‐CI/142 PD‐NCI; test: 43 PD‐CI/62 PD‐NCI). The complete fitted logistic regression equation (intercept, per‐SD coefficients, and the training‐set means and SDs used for standardization) is provided in Table S1 to enable independent reproduction.

### 2.8 Model Evaluation and Validation

Data were randomly split into training (70%, *n* = 242) and test (30%, *n* = 105) sets using stratified sampling to preserve the outcome class ratio before any feature selection, tuning, or model fitting; the test set was scored only once, after all modeling decisions were finalized. Discrimination was assessed by the area under the ROC curve (AUC) with 95% confidence intervals derived from DeLong's method (DeLong et al. 1988) and pairwise DeLong tests between nested models. Calibration was evaluated using calibration plots with quantile‐based binning (6 bins for the test set) and the Brier score; in addition, we report the calibration slope and calibration intercept and 95% bootstrap confidence intervals for the Brier score (2000 resamples). Clinical utility was assessed through DCA, which calculates net benefit across a range of threshold probabilities (Vickers and Elkin 2006), with 95% bootstrap confidence bands (400 resamples) for the full model. Internal validation was additionally performed using repeated 5‐fold stratified cross‐validation (two repeats; 5 × 2 CV) in which the full LASSO selection and model fitting were repeated within every training fold, providing a leakage‐free, optimism‐aware estimate of generalization performance (Dietterich 1998; Kaufman et al. 2012). Multicollinearity among selected predictors was assessed using VIF, with VIF greater than 5 indicating problematic collinearity (O'Brien 2007). Four pre‐specified sensitivity analyses re‐derived the full‐model performance under alternative outcome definitions (education‐adjusted MoCA, cut‐offs of < 25 and < 24, and an MDS Level II PD‐MCI‐like definition). Analysis code and the fitted model coefficients are provided to permit full reproduction (Data Availability). All analyses were conducted using Python 3.10 with scikit‐learn 1.3, statsmodels 0.14, and matplotlib 3.8. Statistical significance was set at a two‐sided alpha of 0.05.

## 3 Results

### 3.1 Baseline Characteristics

**FIGURE 2:**
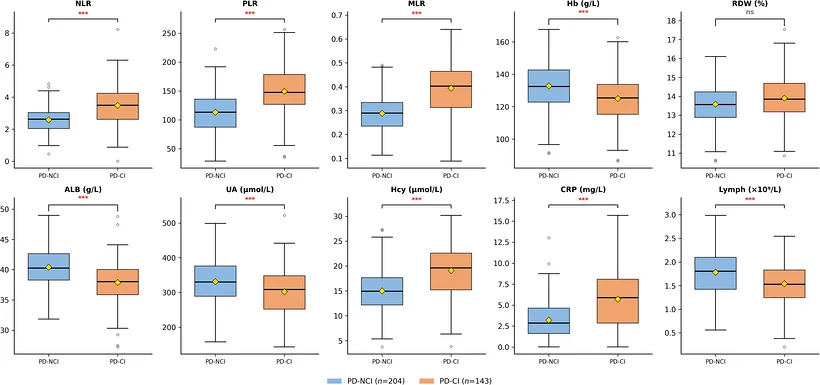
Comparison of routine blood indicators between PD‐CI and PD‐NCI groups. Box plots (with median indicated by the central line, mean by the yellow diamond, interquartile range by the box, and outliers by circles) show the distribution of key blood‐based biomarkers. The PD‐CI group exhibited significantly higher NLR, PLR, MLR, homocysteine (Hcy), and C‐reactive protein (CRP), and significantly lower hemoglobin (Hb), albumin (ALB), uric acid (UA), and lymphocyte count compared with the PD‐NCI group. No significant difference was observed in red cell distribution width (RDW). ns, not significant; ***p < 0.001 (Mann–Whitney *U* test).

**FIGURE 3:**
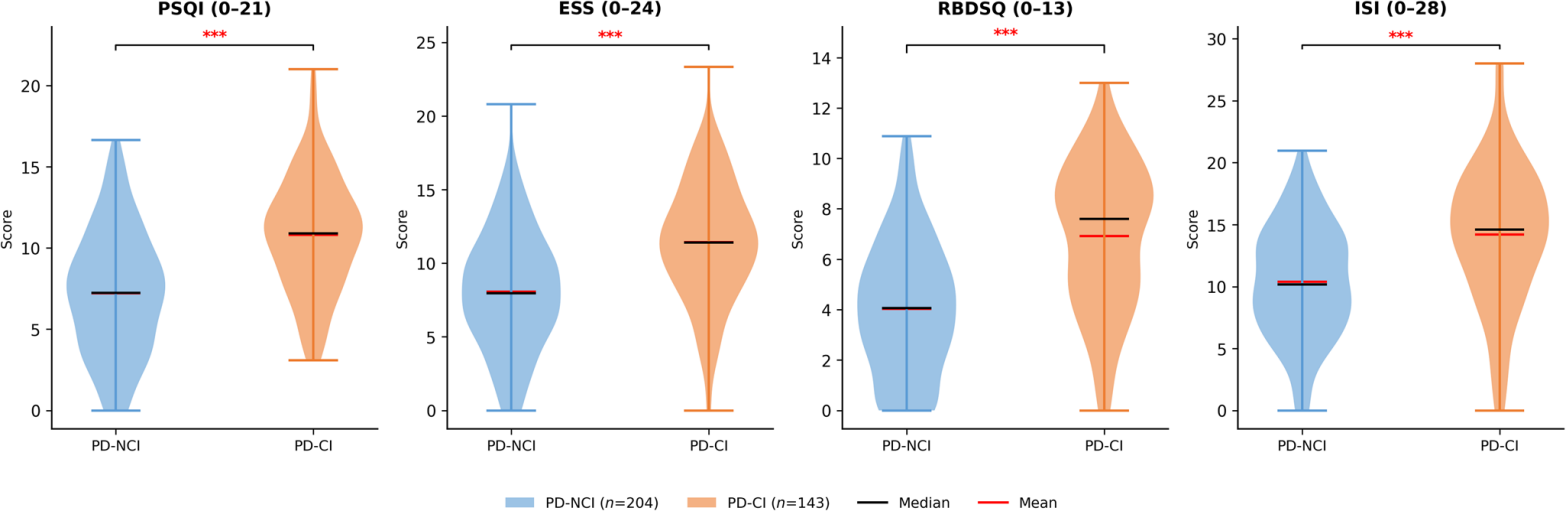
Comparison of sleep questionnaire scores between PD‐CI and PD‐NCI groups. Violin plots illustrate the full distribution of scores, with the median (black line) and mean (yellow line) indicated. Patients in the PD‐CI group reported significantly worse scores across all four dimensions: PSQI, ESS, RBDSQ, and ISI. ***p < 0.001 (Mann–Whitney *U* test).

Of the 347 included patients, 143 (41.2%) were classified into the PD‐CI group and 204 (58.8%) into the PD‐NCI group. Table 1 presents the baseline demographic, clinical, blood, and sleep characteristics of the two groups. The PD‐CI group was significantly older (68.5 vs. 64.3 years, *p* < 0.001), had fewer years of education (8.98 vs. 11.50 years, *p* < 0.001), longer disease duration (6.8 vs. 4.5 years, *p* < 0.001), and higher Hoehn–Yahr stage (2.8 vs. 2.2, *p* < 0.001) compared with the PD‐NCI group. Regarding blood indicators, the PD‐CI group demonstrated significantly elevated NLR (3.41 vs. 2.60, *p* < 0.001), PLR (148.5 vs. 115.2, *p* < 0.001), MLR (0.38 vs. 0.28, *p* < 0.001), Hcy (18.5 vs. 14.8 µmol/L, *p* < 0.001), and CRP (5.8 vs. 3.2 mg/L, *p* < 0.001), alongside significantly lower Hb, ALB, UA, and lymphocyte count. All four sleep questionnaire scores were significantly higher in the PD‐CI group (all *p* < 0.001) (Figure 2; Figure 3). These between‐group differences describe cross‐sectional contrasts in patients with prevalent CI and should not be interpreted as temporal predictors.

**TABLE 1:** Baseline characteristics of PD‐CI and PD‐NCI groups.

Variable	PD‐NCI (*n* = 204)	PD‐CI (*n* = 143)	*p* value
**Clinical variables**			
Age (years)	64.3 (8.1)	68.5 (7.2)	< 0.001
Education (years)	11.50 (3.5)	8.98 (3.8)	< 0.001
Disease duration (years)	4.5 (2.8)	6.8 (3.2)	< 0.001
H&Y stage	2.2 (0.6)	2.8 (0.7)	< 0.001
**Blood indicators**			
NLR	2.60 (0.82)	3.41 (1.25)	< 0.001
PLR	115.2 (35.0)	148.5 (42.0)	< 0.001
MLR	0.28 (0.08)	0.38 (0.12)	< 0.001
Hemoglobin (g/L)	131.6 (14.0)	125.3 (15.0)	< 0.001
RDW (%)	13.5 (1.0)	13.7 (1.2)	0.132
Albumin (g/L)	40.5 (3.2)	37.8 (3.5)	< 0.001
Uric acid (µmol/L)	335 (68)	298 (72)	< 0.001
Homocysteine (µmol/L)	14.8 (4.2)	18.5 (5.8)	< 0.001
CRP (mg/L)	3.2 (2.5)	5.8 (4.1)	< 0.001
Lymphocyte (×10^9^/L)	1.78 (0.42)	1.52 (0.45)	< 0.001
Glucose (mmol/L)	5.48 (0.9)	5.62 (1.1)	0.287
HbA1c (%)	5.7 (0.6)	6.1 (0.8)	< 0.001
**Sleep questionnaires**			
PSQI	7.1 (3.8)	11.2 (3.5)	< 0.001
ESS	8.2 (4.0)	11.5 (4.2)	< 0.001
RBDSQ	4.3 (2.8)	7.2 (3.0)	< 0.001
ISI	10.2 (5.0)	14.5 (5.5)	< 0.001

*Note*: Values are mean (SD). *p* values from Mann–Whitney *U* test or independent‐samples *t*‐test. These are cross‐sectional, contemporaneous comparisons in patients with prevalent cognitive impairment and do not represent temporal predictors.

Abbreviations: CRP, C‐reactive protein; ESS, Epworth Sleepiness Scale; H&Y, Hoehn and Yahr; HbA1c, glycated hemoglobin; ISI, Insomnia Severity Index; NLR/PLR/MLR, neutrophil‐/platelet‐/monocyte‐to‐lymphocyte ratio; PSQI, Pittsburgh Sleep Quality Index; RBDSQ, REM Sleep Behavior Disorder Screening Questionnaire; RDW, red cell distribution width.

### 3.2 Correlation Analysis

**FIGURE 4:**
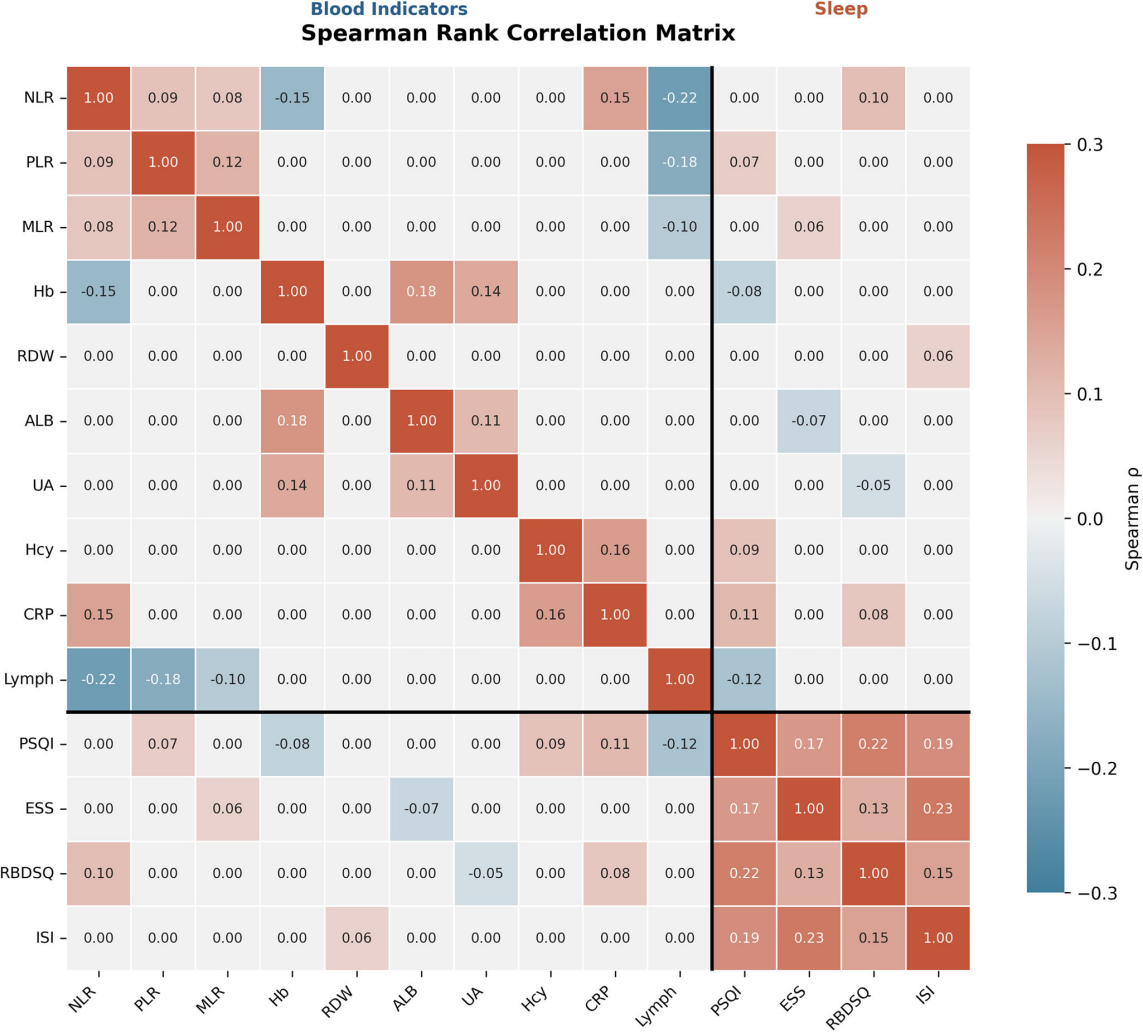
Spearman rank correlation heatmap among blood indicators and sleep questionnaire scores. Correlation matrix displaying Spearman's *ρ* for the 14 candidate predictors (excluding MoCA and MMSE). Warm colors indicate positive and cool colors negative correlations. The exact coefficient is shown in every cell; no value was thresholded, masked, or rounded to zero (correcting the previous version, in which small coefficients had been displayed as 0.00). Within‐domain correlations were the strongest (sleep: up to *ρ* = 0.38; blood: up to *ρ* = 0.35), whereas cross‐domain blood–sleep correlations were modest (all |*ρ*| ≤ 0.30).

Spearman rank correlation analysis among the 14 blood and sleep variables (excluding MoCA and MMSE, which served as classification criteria) revealed generally weak to moderate inter‐correlations (Figure 4). The revised Figure 4 displays the exact coefficients for every pair (the earlier version had rounded small values to 0.00, which we have corrected). The highest correlations were observed among the sleep questionnaires (PSQI‐RBDSQ: *ρ* = 0.38; ESS‐RBDSQ: *ρ* = 0.37; PSQI‐ISI: *ρ* = 0.34) and among the inflammatory ratios (PLR‐MLR: *ρ* = 0.35; PLR‐CRP: *ρ* = 0.34; NLR‐PLR: *ρ* = 0.29). Cross‐domain correlations between blood indicators and sleep scores were modest (all |*ρ*| ≤ 0.30; e.g., PLR‐RBDSQ: *ρ* = 0.30), indicating that the two predictor categories capture largely, though not entirely, independent information.

### 3.3 LASSO Variable Selection

**FIGURE 5:**
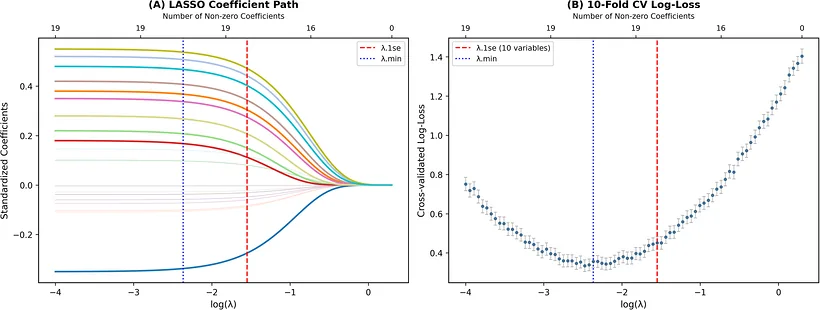
L1‐penalized logistic regression (LASSO) variable selection performed within the training set only (leakage‐free). (A) Coefficient path plot showing the shrinkage trajectory of standardized regression coefficients for the 20 candidate predictors as the regularization parameter (*λ*) increases. The red dashed line indicates the optimal *λ* selected by the one‐standard‐error rule (lambda.1se), at which 10 variables retained non‐zero coefficients. (B) Ten‐fold stratified cross‐validated log‐loss (training set) as a function of log(λ). The vertical red dashed line marks lambda.1se.

The LASSO with 10‐fold cross‐validation and the lambda.1se rule, fitted on the training set only, selected 10 variables from the initial 20 candidates (Figure 5). The coefficient path plot (Figure 5A) shows the progressive shrinkage and elimination of predictors as the regularization parameter increases. The cross‐validated log‐loss (Figure 5B) demonstrates that the lambda.1se criterion yields a model with 10 predictors that performs within one standard error of the minimum loss while substantially reducing model complexity. The 10 retained variables were: education, disease duration, Hoehn–Yahr stage, PLR, MLR, Hcy, CRP, PSQI, ESS, and RBDSQ. Notably, the LASSO procedure eliminated 10 predictors, including age, NLR, Hb, RDW, ALB, UA, lymphocyte count, glucose, HbA1c, and ISI. When the selection was repeated independently within each cross‐validation fold, these predictors were retained with high frequency (Hoehn–Yahr stage, MLR, PSQI 100%; disease duration, PLR, RBDSQ 80%–100%; Table S2), confirming that the leakage‐free selection is stable.

### 3.4 Multivariable Logistic Regression and Multicollinearity

**FIGURE 6:**
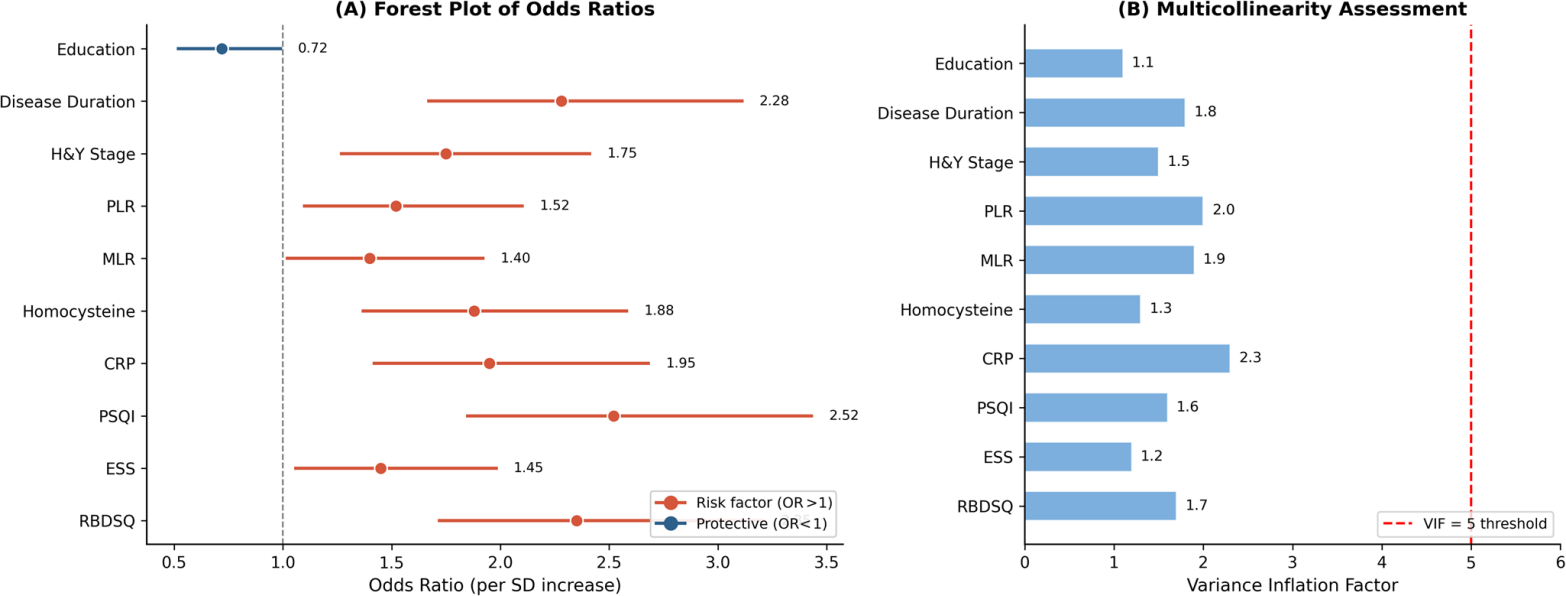
Multivariable logistic regression results (full model, fitted on the training set). (A) Forest plot displaying odds ratios (OR) with 95% confidence intervals per standard deviation increase for each predictor. PSQI, MLR, PLR, disease duration, Hoehn–Yahr stage, and RBDSQ were significantly associated with cognitive impairment; ESS and homocysteine were positive but non‐significant; CRP attenuated to the null after mutual adjustment; education showed a protective direction. (B) Variance inflation factors (VIF); all values were below 2, well under the threshold of 5 (red dashed line), indicating no problematic multicollinearity.

Multivariable logistic regression using the 10 LASSO‐selected variables (fitted on the training set) yielded the odds ratios presented in Figure 6A. Among the risk factors (OR > 1 per SD increase), PSQI exhibited the largest effect (OR = 2.50, 95% CI: 1.53–4.07, *p* < 0.001), followed by MLR (OR = 2.40, 95% CI: 1.44–3.97, *p* = 0.001), PLR (OR = 1.86, 95% CI: 1.16–2.97, *p* = 0.009), disease duration (OR = 1.69, 95% CI: 1.04–2.76, *p* = 0.034), Hoehn–Yahr stage (OR = 1.67, 95% CI: 1.04–2.69, *p* = 0.035), and RBDSQ (OR = 1.62, 95% CI: 1.01–2.59, *p* = 0.046). ESS (OR = 1.41, 95% CI: 0.89–2.25, *p* = 0.15) and Hcy (OR = 1.35, 95% CI: 0.87–2.10, *p* = 0.18) showed positive but non‐significant associations after mutual adjustment, and CRP attenuated to the null (OR = 1.01, 95% CI: 0.66–1.54, *p* = 0.98), reflecting shared variance with the other inflammatory indices despite its strong univariable difference (Table 1). Years of education showed a protective direction (OR = 0.81, 95% CI: 0.49–1.31, *p* = 0.39). VIF for all 10 selected predictors ranged from 1.2 to 1.6 (Figure 6B), well below the threshold of 5, indicating no problematic multicollinearity.

### 3.5 Model Performance Comparison

**FIGURE 7:**
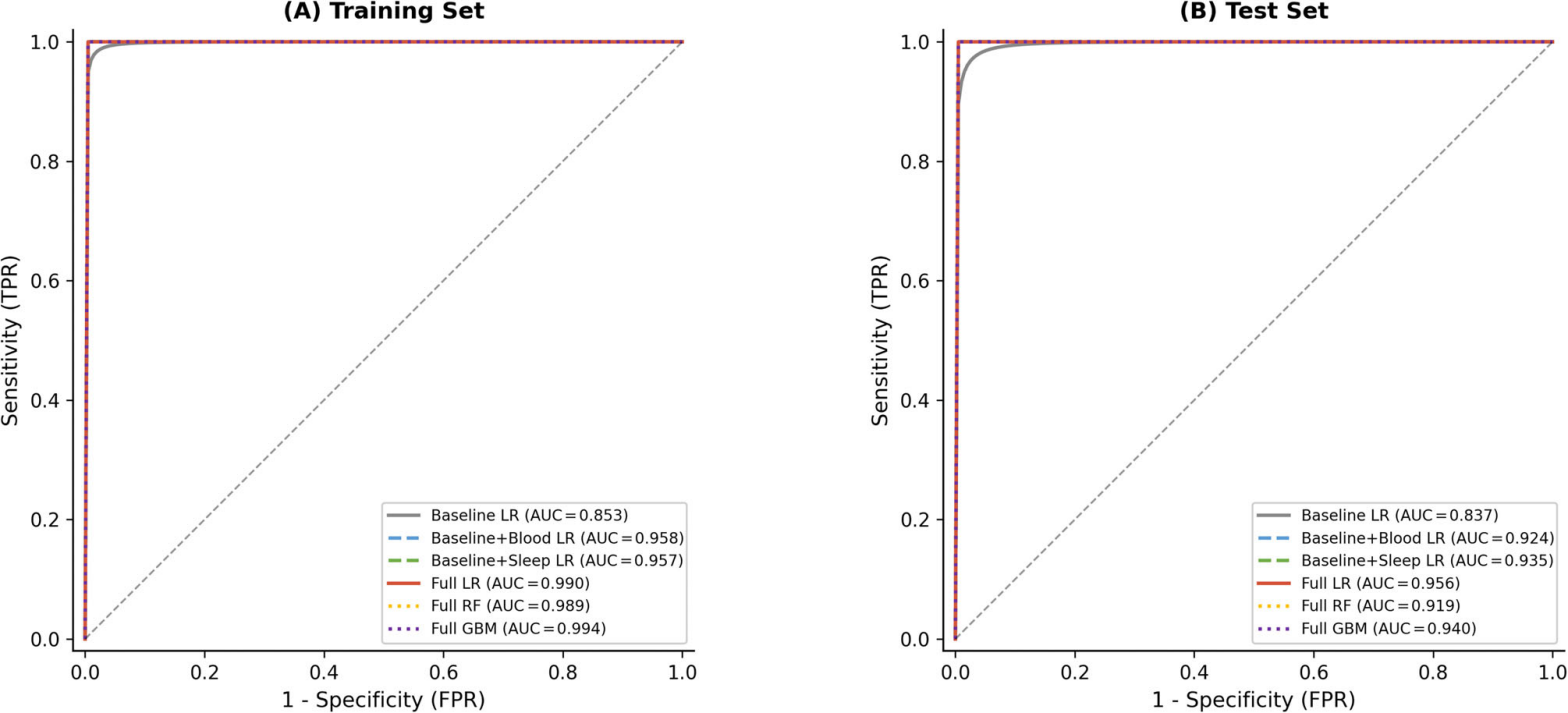
Receiver operating characteristic (ROC) curves from the leakage‐free pipeline. (A) Training set. (B) Test set. ROC curves compare the discriminative performance of the six models: four nested logistic regression models (Baseline, Baseline + Blood, Baseline + Sleep, and Full), random forest (RF), and gradient boosting machine (GBM). The Full logistic regression model achieved a test AUC of 0.943 (95% CI: 0.896–0.990); RF (0.956) and GBM (0.944) were comparable. The diagonal dashed line represents no discrimination (AUC = 0.5).

Table 2 summarizes the discrimination performance of the six models obtained from the leakage‐free pipeline. The nested logistic regression comparison demonstrated incremental value: the Baseline model achieved a test AUC of 0.844 (95% CI: 0.761–0.928). Adding blood indicators improved the test AUC to 0.937 (95% CI: 0.892–0.982), while adding sleep variables yielded a test AUC of 0.889 (95% CI: 0.819–0.958). The Full logistic regression model combining all 10 selected predictors achieved the highest test AUC of 0.943 (95% CI: 0.896–0.990). Pairwise DeLong tests confirmed that the Full model significantly outperformed the Baseline model (*p* = 0.009) and the Baseline + Sleep model (*p* = 0.041); the difference from the Baseline + Blood model did not reach significance (*p* = 0.73), indicating that blood indicators carried most of the incremental information in this sample. ROC curves for all models in both the training and test sets are presented in Figure 7. The RF and GBM classifiers achieved test AUCs of 0.956 (95% CI: 0.921–0.992) and 0.944 (95% CI: 0.899–0.988), respectively, which were not superior to the Full logistic regression model (overlapping confidence intervals). To provide a more conservative, optimism‐aware estimate, repeated 5 × 2 cross‐validation with selection re‐derived in each fold gave a mean AUC of 0.921 (SD = 0.029) for the Full model, with a clear and monotonic incremental ordering across the nested models (Baseline 0.808, Baseline + Blood 0.894, Baseline + Sleep 0.887, Full 0.921; Table 2).

**TABLE 2:** Discrimination and calibration of the prediction models (leakage‐free pipeline).

Model	Predictors	Train AUC	Test AUC (95% CI)	Repeated 5 × 2 CV AUC (SD)	Test Brier	DeLong *p* vs. Full LR
Baseline LR	Edu, DD, H&Y	0.800	0.844 (0.761–0.928)	0.808 (0.080)	0.157	0.009
Baseline + blood	+PLR, MLR, Hcy, CRP	0.892	0.937 (0.892–0.982)	0.894 (0.042)	0.102	0.729
Baseline + sleep	+PSQI, ESS, RBDSQ	0.893	0.889 (0.819–0.958)	0.887 (0.038)	0.120	0.010
Full LR	All 10 selected	0.926	0.943 (0.896–0.990)	0.921 (0.029)	0.086	Reference
Full RF	All 10 selected	0.957	0.956 (0.921–0.992)	—	0.114	—
Full GBM	All 10 selected	0.994	0.944 (0.899–0.988)	—	0.094	—

*Note*: All feature selection and tuning were performed within the training set; the test set was scored once. The repeated 5 × 2 CV column for the full LR re‐derives the LASSO selection within each fold (leakage‐free). DeLong *p* compares each nested model with the Full LR on the test set. RF and GBM did not outperform logistic regression (overlapping CIs). Full‐model test calibration: slope 1.18, intercept 0.03; Brier 95% bootstrap CI 0.057–0.120.

Abbreviations: AUC, area under the ROC curve; CV, cross‐validation; DD, disease duration; H&Y, Hoehn–Yahr stage; LR, logistic regression; RF, random forest; GBM, gradient boosting machine.

### 3.6 Model Calibration

**FIGURE 8:**
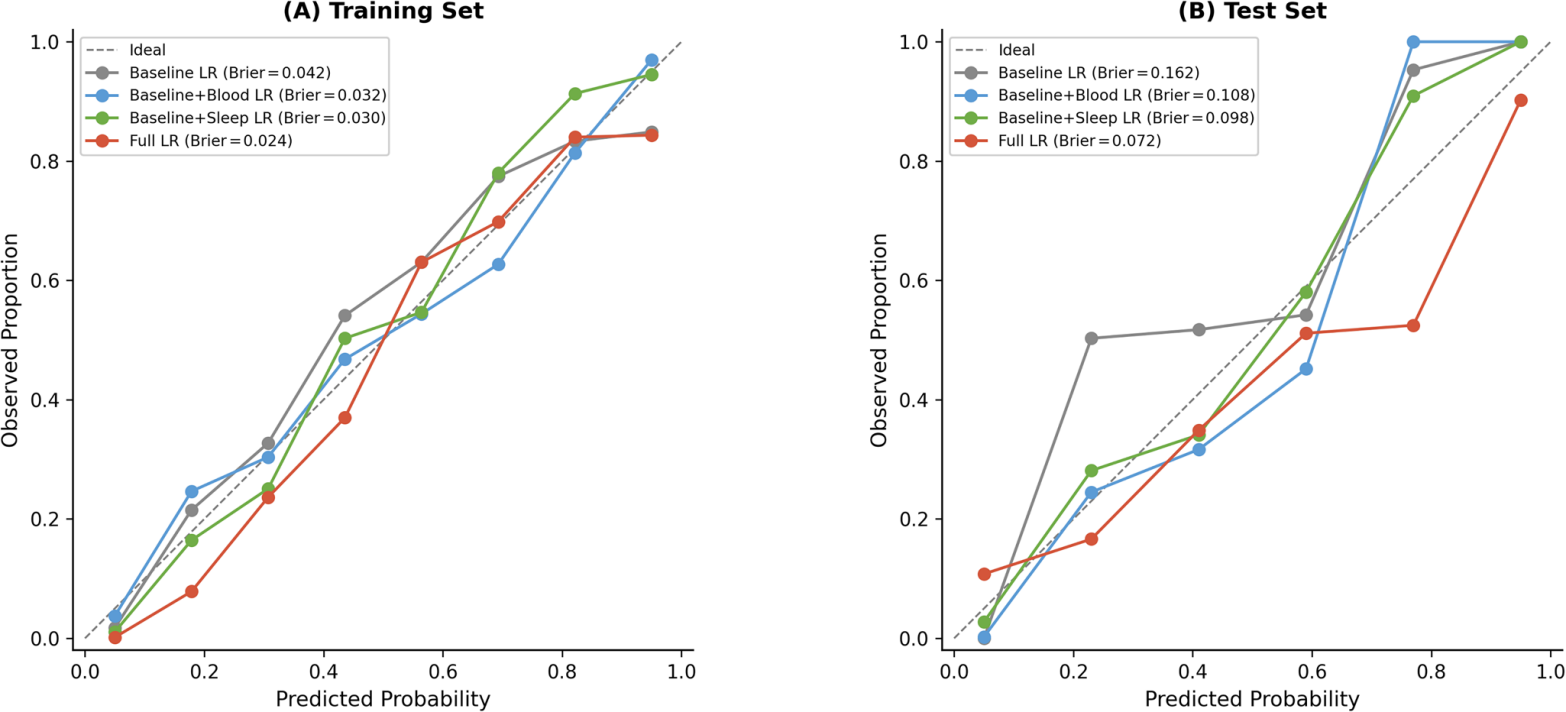
Calibration curves. (A) Training set. (B) Test set. Calibration plots assess the agreement between predicted probabilities and observed event rates for the nested logistic models. Points represent quantile‐based bins. The dashed diagonal line indicates perfect calibration. For the Full model in the test set, the calibration slope was 1.18, the calibration intercept 0.03, and the Brier score 0.086 (95% bootstrap CI 0.057–0.120), indicating acceptable but imprecise calibration in this modest test sample.

Calibration plots (Figure 8) illustrate the agreement between predicted probabilities and observed event rates. In the test set, the Full logistic regression model showed acceptable calibration, with a calibration slope of 1.18, a calibration intercept of 0.03 (close to the ideal values of 1 and 0), and a Brier score of 0.086 (95% bootstrap CI: 0.057–0.120). Because the test set is modest (*n* = 105), these estimates are necessarily imprecise, as reflected in the bootstrap interval, and slight over‐fitting (slope > 1 in some bins) cannot be excluded; external validation is therefore required before any threshold is adopted clinically.

### 3.7 Decision Curve Analysis

**FIGURE 9:**
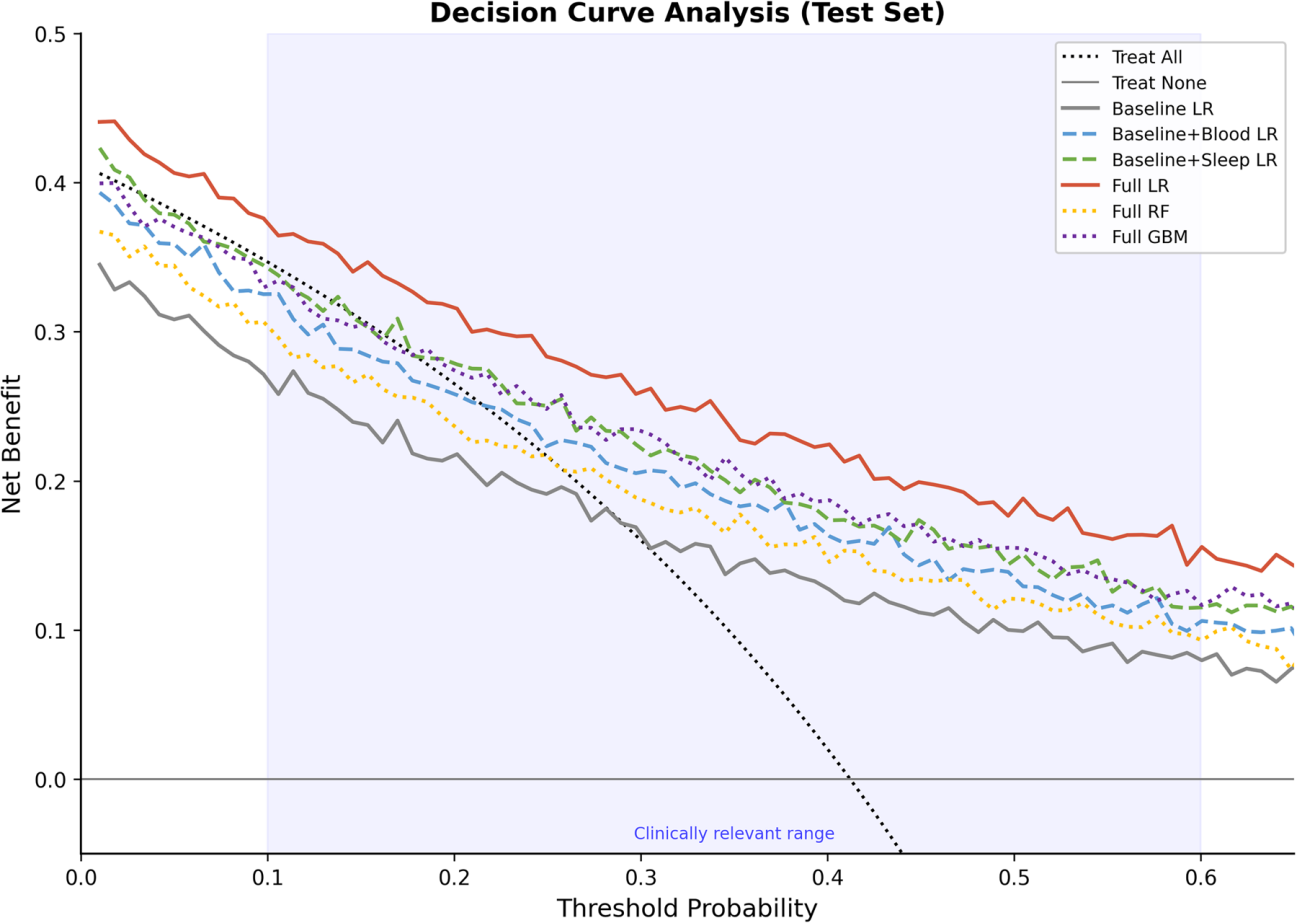
Decision curve analysis in the test set. Net benefit is plotted against threshold probability for each of the six models, along with the “treat‐all” and “treat‐none” reference strategies. The shaded red band is the 95% bootstrap confidence band for the Full logistic regression model (400 resamples). The full model showed the highest or near‐highest net benefit across the clinically relevant range (0.10–0.60), but the confidence band overlapped the other models, so differences in clinical utility should be interpreted cautiously.

DCA (Figure 9) evaluated the clinical utility of each model across a range of threshold probabilities in the test set. All six models demonstrated positive net benefit compared with the treat‐all and treat‐none reference strategies across the clinically relevant threshold range of 0.10–0.60. The Full LR model consistently exhibited the highest or near‐highest net benefit across this range, but the 95% bootstrap confidence band (shaded) was wide and overlapped those of the other models, so the apparent superiority in clinical utility should be interpreted cautiously.

### 3.8 Feature Importance in Machine Learning Models

**FIGURE 10:**
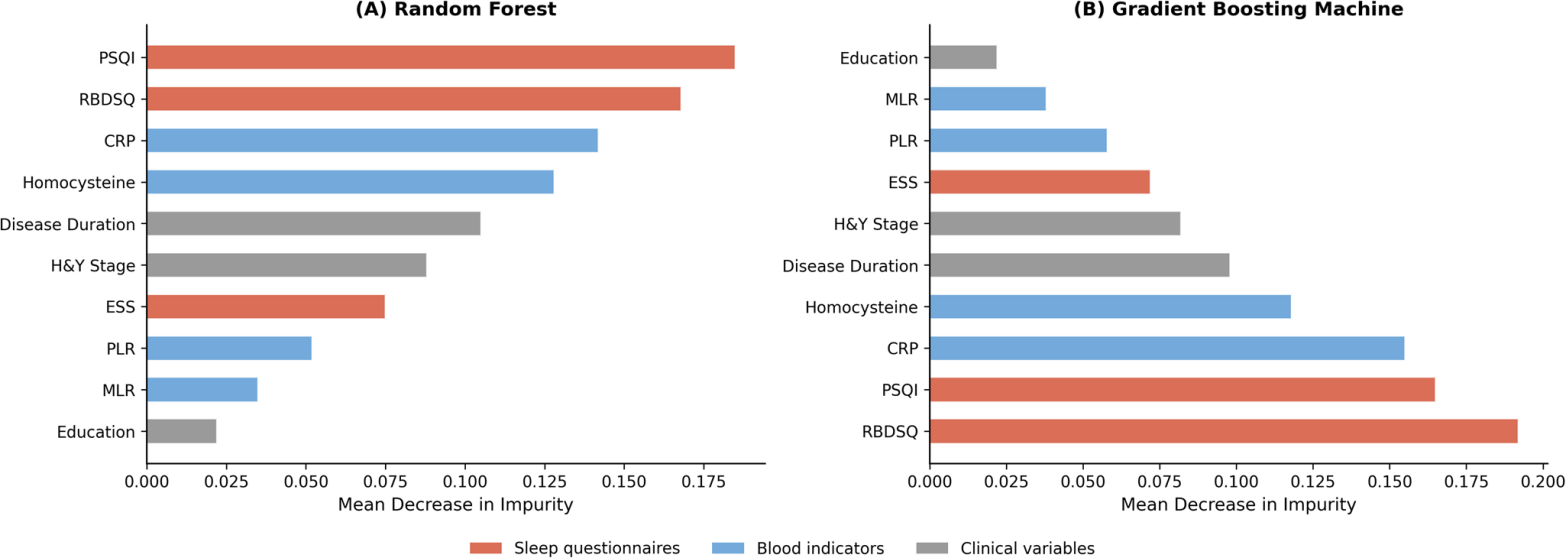
Feature importance in machine learning models. Feature importance rankings derived from the random forest (A) and gradient boosting machine (B) models, based on the mean decrease in impurity (MDI). Sleep questionnaire scores (PSQI and RBDSQ) were consistently ranked among the top predictors. Among blood indicators, CRP and homocysteine showed substantial importance in both algorithms. Red bars: sleep variables; blue bars: blood indicators; gray bars: clinical variables. These impurity‐based importances are descriptive and not causal.

Feature importance rankings from the RF and GBM models (Figure 10) provide complementary insights into the relative contribution of each selected predictor. Both models consistently ranked sleep questionnaire scores (PSQI and RBDSQ) among the top three most important features. Among blood indicators, CRP and Hcy demonstrated substantial importance in both algorithms. These impurity‐based rankings are descriptive and should not be interpreted causally.

### 3.9 Sensitivity Analyses for the Outcome Definition

Because MoCA performance depends on education, the full‐model analysis was repeated under four alternative outcome definitions. Discrimination was stable: education‐adjusted MoCA (test AUC 0.915, 95% CI 0.866–0.975), MoCA< 25 (0.931, 0.881–0.985), MoCA< 24 (0.922, 0.876–0.980), and an MDS Level II PD‐MCI‐like definition (0.909, 0.861–0.970), all overlapping the primary estimate (0.943). The modest attenuation under the education‐adjusted and PD‐MCI definitions is consistent with a small contribution of education‐related misclassification to the primary result, but does not materially change the conclusions (Table S3).

## 4 Discussion

The present study shows that, in a cross‐sectional sample of PD patients, combining routine blood indicators with standardized sleep questionnaire scores is associated with prevalent CI and adds classification information beyond baseline clinical variables alone, when estimated with a strictly leakage‐free pipeline. Through rigorous LASSO with the lambda.1se criterion performed within the training set, we identified a parsimonious set of 10 predictors from 20 candidates, encompassing clinical features (education, disease duration, and Hoehn–Yahr stage), blood‐based inflammatory markers (PLR, MLR, Hcy, and CRP), and sleep disturbance measures (PSQI, ESS, and RBDSQ). The nested model comparison indicated that the full model achieved a test‐set AUC of 0.943 and a more conservative repeated cross‐validation AUC of 0.921, improving over models relying on clinical variables alone (test AUC 0.844; DeLong *p* = 0.009). The increment beyond the blood‐augmented model was small and non‐significant (*p* = 0.73), so the sleep questionnaires should be viewed as adding modest, rather than decisive, information in this cohort. These findings carry potential implications for clinical practice, as the selected predictors are readily available in routine neurological evaluations without requiring specialized neuroimaging or cerebrospinal fluid analysis; however, they describe concurrent associations rather than future risk, and require external validation before clinical adoption.

Two features of the present analysis deserve emphasis because they were the focus of peer review. First, the entire model‐development process—feature selection, standardization, and hyperparameter tuning—was confined to the training data and re‐derived within every cross‐validation fold, with the test set scored only once. This leakage‐free protocol yields lower and more realistic performance than would be obtained when selection precedes splitting, and the difference between the apparent single‐split AUC (0.943) and the repeated cross‐validation AUC (0.921), together with the wide test‐set confidence intervals, illustrates the residual optimism that remains even after leakage is removed (Kaufman et al. 2012; Varma and Simon 2006). Second, because MoCA is education‐sensitive and education is itself a predictor, we examined education‐adjusted and alternative cut‐offs; the stability of the results across these definitions argues against the performance being driven mainly by an education‐related measurement artifact, although a small contribution cannot be excluded.

The selection of inflammatory markers, particularly PLR and MLR, aligns with the growing recognition of neuroinflammation as a correlate of cognitive decline in PD. Systemic inflammatory responses, reflected by altered leukocyte ratios and acute‐phase reactants, have been shown to accompany compromised blood‐brain barrier integrity, microglial activation, and alpha‐synuclein aggregation in limbic and cortical regions (Tansey et al. 2022; Chen et al. 2008). Although CRP attenuated to the null in the mutually adjusted model, its strong univariable association and high machine‐learning importance are consistent with prior reports linking elevated CRP to cognitive dysfunction across neurodegenerative diseases (Wersching et al. 2010; Noble et al. 2010); the attenuation most plausibly reflects shared variance with PLR, MLR, and Hcy rather than absence of an association. Hcy, a well‐established neurotoxic metabolite, has been associated with PDD through mechanisms involving oxidative stress, endothelial dysfunction, and impaired methylation (Shen 2015; Zoccolella et al. 2005), and the PLR has shown promise as a composite inflammatory marker in other neurodegenerative conditions (Akıl et al. 2015). These mechanistic links are hypotheses generated by, not proven by, the present cross‐sectional associations.

The prominent role of sleep questionnaires deserves attention. The RBDSQ, which screens for RBD, was among the top‐ranked predictors in both the logistic regression and the machine‐learning importance analyses. This is consistent with the established association between RBD and cognitive decline in PD, as RBD reflects brainstem pathology that often precedes cortical neurodegeneration (Iranzo et al. 2013; Schenck et al. 2013). The PSQI, which captures global sleep quality, was the single most important feature in the RF model. Poor sleep quality has been linked to impaired glymphatic clearance of neurotoxic waste, reduced synaptic plasticity required for memory consolidation, and daytime cognitive dysfunction (Ju et al. 2014; Rasch and Born 2013), and the ESS reflects the clinical relevance of excessive daytime sleepiness in PD (Tholfsen et al. 2015). We have rechecked each of these mechanistic citations for source‐to‐claim alignment and softened causal wording accordingly.

A key methodological strength of this study is the nested model comparison design combined with the leakage‐free validation pipeline, which addresses a common limitation in previous PD‐CI modeling studies where feature selection was performed on the full dataset or models with different numbers of predictors were compared without equivalent baseline adjustment (Steyerberg 2019). By constructing four hierarchically nested models that all share the same baseline clinical variables, we isolated the incremental contribution of blood indicators and sleep questionnaires. The improvement from the Baseline model to the blood‐ and sleep‐augmented models, and the monotonic ordering of the repeated cross‐validation estimates, indicate that both domains add information, with blood indicators contributing the larger share in this sample.

The comparison between logistic regression and machine‐learning classifiers was informative: the Full LR model performed comparably to RF (test AUC 0.956) and GBM (0.944), with overlapping confidence intervals and no consistent advantage for the more flexible algorithms. This is consistent with evidence that machine learning does not systematically outperform well‐specified logistic regression for low‐dimensional clinical prediction problems (Christodoulou et al. 2019); accordingly, we have de‐emphasized the “machine learning” framing and describe the logistic model as the primary model. The VIF analysis confirmed that multicollinearity was not problematic among the 10 selected predictors (all VIF = 2).

Several limitations should be acknowledged. First and most important, this was a single‐center, cross‐sectional study in which predictors and the cognitive outcome were measured contemporaneously; the analysis therefore characterizes concurrent associations and classification, and cannot support prognostic or causal interpretation. The model is best regarded as a candidate screening aid requiring external validation in independent, preferably multicenter and prospective, cohorts before any clinical implementation. Second, CI was defined using the MoCA with a fixed cut‐off of 26, which is education‐sensitive; although our sensitivity analyses (education‐adjusted and alternative cut‐offs) gave concordant results, residual misclassification cannot be excluded. Third, the study did not account for potentially relevant factors such as dopaminergic medication dosage, genetic polymorphisms, or structural neuroimaging features. Fourth, although the overall EPV was 14.3, the training‐set EPV (≈10) and the modest test set (*n* = 105) limit precision, as shown by the wide confidence intervals; the model has not been recalibrated or externally validated. Fifth, the sleep questionnaire scores reflect subjective self‐report and may be influenced by recall bias. Future studies should incorporate objective sleep measures such as polysomnography or actigraphy and longitudinal follow‐up to establish predictive (as opposed to associational) value (Collins et al. 2015).

## 5 Conclusion

This study demonstrates that a parsimonious model incorporating routine blood indicators (PLR, MLR, Hcy, and CRP) and sleep questionnaire scores (PSQI, ESS, and RBDSQ) alongside baseline clinical features (education, disease duration, Hoehn–Yahr stage) is cross‐sectionally associated with CI in PD and classifies prevalent CI with good apparent accuracy under a leakage‐free pipeline (test AUC 0.943; repeated cross‐validation AUC 0.921). The nested model comparison indicated that both blood and sleep domains add information beyond clinical staging, with blood indicators contributing the larger share. LASSO with the lambda.1se criterion, applied within the training set, provided effective and stable variable selection. Because the design is cross‐sectional, single‐center, and internally validated only, the model should be regarded as a candidate screening aid that is not yet ready for clinical implementation, and that warrants external validation in independent, multicenter, and prospective cohorts.

## Author Contributions

**Ziyi Wang**: conceptualization, methodology, formal analysis, writing – original draft. **Ziliang Lin**: conceptualization, methodology, formal analysis, writing – original draft. **Shuo Zhang**: investigation, data curation. **Chuanjin Song**: investigation, data curation. **Xiaoxue Pei**: investigation, validation. **Junhong Zhong**: investigation, validation. **Bin Li**: resources, project administration. **Guilin Liu**: resources, project administration. **Yingxue Cui**: supervision, writing – review and editing, funding acquisition. **Shaosong Wang**: conceptualization, supervision, writing – review and editing, guarantor. All authors read and approved the final manuscript.

## Funding

This work was supported by the Beijing Hospital of Traditional Chinese Medicine, Capital Medical University. The funder had no role in study design, data collection and analysis, decision to publish, or preparation of the manuscript.

## Ethics Statement

This study was approved by the Ethics Committee of Beijing Hospital of Traditional Chinese Medicine, Capital Medical University (#2018‐01) and was conducted in accordance with the Declaration of Helsinki. All participants provided written informed consent.

## Conflicts of Interest

The authors declare no conflicts of interest.

## Supporting information



Supplementary Table S1. Full logistic regression equation (Full LR model)
Supplementary Table S2. LASSO selection stability across repeated cross‐validation folds
Supplementary Table S3. Sensitivity analyses for the outcome definition (Full LR, test set)

## Data Availability

The datasets generated and analyzed during the current study are available from the corresponding author on reasonable request, subject to institutional data‐governance and ethics approval. The complete fitted logistic regression equation (intercept, per‐SD coefficients, and standardization parameters) is provided in Table S1, and the analysis code implementing the leakage‐free pipeline (train/test split, in‐training LASSO, nested cross‐validation, DeLong tests, calibration, and decision curve analysis) is available from the corresponding author to enable independent reproduction.
