# Supplementary material for: Routine Blood Indicators Combined With Sleep Questionnaires for the Identification of Cognitive Impairment in Parkinson's Disease: A Cross‐Sectional Study With a Leakage‐Free Nested Modeling Framework
Source: Brain Behav. 2026 Sep 7;16(9):e71655. doi: 10.1002/brb3.71655 (PMC13550880; doi:10.1002/brb3.71655)
Supplement: Supplementary file 1 [file BRB3-16-e71655-s001.docx]

**Supplementary Materials**

*Manuscript 1424183 — Brain and Behavior*

**Supplementary Table S1. Full logistic regression equation (Full LR model)**

The predicted log-odds of cognitive impairment is: logit(p) = β0 + Σ βi × (xi − meani)/SDi, where xi are the raw predictor values and (meani, SDi) are the TRAINING-set standardization parameters. Coefficients are per 1-SD increase.

| **Term** | **β (per SD)** | **Training mean** | **Training SD** | **Odds ratio (95% CI)** |
| --- | --- | --- | --- | --- |
| Intercept (β0) | -0.6205 | — | — | — |
| Education (years) | -0.2168 | 10.293 | 3.850 | 0.81 (0.49–1.31) |
| Disease duration (years) | +0.5267 | 5.439 | 3.215 | 1.69 (1.04–2.76) |
| H&Y stage | +0.5137 | 2.488 | 0.750 | 1.67 (1.04–2.69) |
| PLR | +0.6202 | 130.429 | 43.831 | 1.86 (1.16–2.97) |
| MLR | +0.8738 | 0.314 | 0.109 | 2.40 (1.44–3.97) |
| Homocysteine (μmol/L) | +0.3006 | 16.407 | 5.148 | 1.35 (0.87–2.10) |
| CRP (mg/L) | +0.0060 | 4.370 | 3.214 | 1.01 (0.66–1.54) |
| PSQI | +0.9153 | 8.693 | 4.018 | 2.50 (1.53–4.07) |
| ESS | +0.3461 | 9.726 | 4.396 | 1.41 (0.89–2.25) |
| RBDSQ | +0.4816 | 5.559 | 3.278 | 1.62 (1.01–2.59) |

*Random seed = 20260612. Training set n=242 (100 PD-CI / 142 PD-NCI); test set n=105 (43 PD-CI / 62 PD-NCI). Predictors standardized with training-set statistics, applied unchanged to the test set. Odds ratios from the multivariable model fitted on the training set.*

**Supplementary Table S2. LASSO selection stability across repeated cross-validation folds**

Selection frequency of each candidate predictor when the entire LASSO procedure (λ.1se) was re-derived independently within each of the 10 cross-validation folds (5-fold × 2 repeats).

| **Candidate** | **Domain** | **Selection frequency** |
| --- | --- | --- |
| Education | Clinical | 40% |
| Disease duration | Clinical | 100% |
| H&Y stage | Clinical | 100% |
| PLR | Blood | 100% |
| MLR | Blood | 100% |
| Homocysteine | Blood | 100% |
| CRP | Blood | 0% |
| PSQI | Sleep | 100% |
| ESS | Sleep | 60% |
| RBDSQ | Sleep | 100% |
| Age | Clinical | 10% |
| NLR | Blood | 100% |
| Hemoglobin | Blood | 40% |
| RDW | Blood | 100% |
| Albumin | Blood | 100% |
| Uric acid | Blood | 70% |
| Lymphocyte | Blood | 100% |
| Glucose | Blood | 90% |
| HbA1c | Blood | 100% |
| ISI | Sleep | 70% |

*The 10 predictors retained in the primary (locked) model were also the most frequently selected under fold-wise re-derivation, confirming that the leakage-free selection is stable.*

**Supplementary Table S3. Sensitivity analyses for the outcome definition (Full LR, test set)**

| **Outcome definition** | **Test AUC (95% CI)** |
| --- | --- |
| MoCA<26 (fixed) | 0.943 (0.896–0.990) |
| MoCA<25 | 0.931 (0.881–0.985) |
| MoCA<24 | 0.922 (0.876–0.980) |
| Education-adjusted MoCA (+1 if ≤6y schooling) | 0.915 (0.866–0.975) |
| MDS PD-MCI Level II-like criteria | 0.909 (0.861–0.970) |

*Education-adjusted MoCA adds one point for participants with ≤6 years of schooling. Discrimination was stable across all definitions, indicating that education-related misclassification did not materially drive the primary result.*
